# Lizards and snakes from the earliest Miocene of Saint-Gérand-le-Puy, France: an anatomical and histological approach of some of the oldest Neogene squamates from Europe

**DOI:** 10.1186/s12862-021-01874-x

**Published:** 2021-07-13

**Authors:** Georgios L. Georgalis, Torsten M. Scheyer

**Affiliations:** University of Zurich, Palaeontological Institute and Museum, Karl Schmid-Strasse 4, 8006 Zurich, Switzerland

**Keywords:** Early Miocene, Taxonomy, Microanatomy, Micro-CT scanning, Lizards, Snakes

## Abstract

**Background:**

The earliest Miocene (Aquitanian) represents a crucial time interval in the evolution of European squamates (i.e., lizards and snakes), witnessing a high diversity of taxa, including an array of extinct forms but also representatives of extant genera. We here conduct a taxonomical survey along with a histological/microanatomical approach on new squamate remains from the earliest Miocene of Saint-Gérand-le-Puy, France, an area that has been well known for its fossil discoveries since the nineteenth century.

**Results:**

We document new occurrences of taxa, among which, the lacertid *Janosikia* and the anguid *Ophisaurus holeci*, were previously unknown from France. We provide a detailed description of the anatomical structures of the various cranial and postcranial remains of lizards and snakes from Saint-Gérand-le-Puy. By applying micro-CT scanning in the most complete cranial elements of our sample, we decipher previously unknown microanatomical features. We report in detail the subsurface distribution and 3D connectivity of vascular channels in the anguid parietal. The fine meshwork of channels and cavities or sinuses in the parietal of *Ophisaurus* could indicate some thermoregulatory function, as it has recently been demonstrated for other vertebrate groups, providing implications for the palaeophysiology of this earliest Miocene anguine lizard.

**Conclusions:**

A combination of anatomical and micro-anatomical/histological approach, aided by micro-CT scanning, enabled the documentation of these new earliest Miocene squamate remains. A distinct geographic expansion is provided for the extinct anguine *Ophisaurus holeci* and the lacertid *Janosikia* (the closest relative of the extant insular *Gallotia* from the Canary Islands).

**Supplementary Information:**

The online version contains supplementary material available at 10.1186/s12862-021-01874-x.

## Background

Our knowledge of the earliest Miocene (Aquitanian) squamates in Europe is limited, with only a few localities yielding, mostly fragmentary, remains of lizards and snakes. As such, Aquitanian squamates from Europe are known from few sporadic finds across a limited number of localities of that age in Germany, France, and Switzerland [1–10]. Complete documentations of Aquitanian European squamate faunas have only been carried out for Amöneburg, Germany (MN 2) [11] and, to a lesser degree, for Ulm, Germany (MN 2) [8, 12–15] and Weisenau, also Germany (MN 1 and/or MN 2) [3–5, 16], and the younger, distinctly less diverse, Bardenas Reales, Spain (MN 2b/3) [17]. A potential Aquitanian squamate fauna has also been briefly described, without any figure, from Oschiri, Sardinia, however, the age of the locality is uncertain and could pertain even to the middle Miocene [18].

This limited number of available earliest Miocene localities hinders significantly our understanding on the evolution, diversity, and taxonomic composition of the oldest Neogene herpetofaunas, especially when considering that the Aquitanian of Europe hosted a diverse array of squamate lineages [11], which became ultimately extirpated or replaced by waves of new immigrant forms from other continents during the Burdigalian, that had a tremendous impact on lizard and snake faunas and reshaped drastically the European herpetofaunal assemblages [19–21]. Such diversity of squamates during the Aquitanian was apparently favored by the paratropical environments that were widespread in Europe and the rises in temperature compared to the preceding late Oligocene [8, 11, 22].

The squamates from the Aquitanian of the Saint-Gérand-le-Puy area, France, have played a significant role in the study of European lizard and snake faunas of that time. Lizard and snake remains from that area have been known since already the middle of the nineteenth century [23], with important, though sporadic, discoveries taking place across the following 150 years [2, 3, 6, 24–27], including also the establishment of new taxa [2, 6, 25]. Vertebrate fossil collections from that area are abundant and scattered across museums and institutions throughout Europe.

Over the past two decades, the use of high resolution (Synchrotron and micro-) computed tomography scanning became a standard and complementary methodology (e.g., [28–30]) to classical osteological or histological studies of extant and extinct animals, including squamates (e.g., [31–33]). Being non-destructive, these modern approaches to study valuable natural history specimens allowed hitherto unprecedented ways of visualizing three-dimensional shapes of tissues and organs, but furthermore revealed also interior morphologies and structures on the histological level. The latter has been used successfully, for example, to reconstruct life history, growth, and skeletochronology data, based on the growth record of hard tissues such as bones and teeth in a wide range of animals. For squamates, specifically postcranial elements (long bones, ribs, vertebrae, and osteoderms) have received much attention by studying their microanatomy and histology (e.g., [34–39]), whereas crania and lower jaws bones remain either little studied [40] or pertain mainly to dental eruption, development, and replacement patterns (e.g., [31, 39, 41–43]; and [44] and references therein).

Here we describe the collection of squamates from the Saint-Gérand-le-Puy area that is curated at the Palaeontological Institute and Museum of the University of Zurich (PIMUZ). We apply micro-CT scanning in the most important specimens, in order to further glean important (micro-)anatomical and histological features from the bones. We also overview in detail the history of squamate discoveries from that area and discuss the biogeographic importance of its earliest Miocene lizard and snake diversity.

## Previous works on squamates from the Saint-Gérand-le-Puy area

The earliest description of squamate remains from the area was made from the site of Langy [23], which is currently considered to pertain to the Saint-Gérand-le-Puy complex [3]. From that locality, three new squamate taxa, i.e., the lizards *Sauromorus ambiguus* Pomel, 1853 and *Sauromorus lacertinus* Pomel, 1853, and the snake *Ophidion antiquus* Pomel, 1853 were coined [23].

Pomel [23] established his new lizard genus *Sauromorus* Pomel, 1853, with the two species *S. ambiguus* and *S. lacertinus* by providing a brief, generalized description, without any figure. The first species, *S. ambiguus* was based on (an imprecisely known number of) specimens from the localities of Langy and Marcouin (currently Marcoin); even the exact nature of the material is not known, but, judging from the description, it should comprise remains of (at least) maxilla(e) and dentary(ies) [23]. The second lizard taxon, *S. lacertinus*, was based on a parietal and some vertebrae from Langy (and not Marcoin, as stated in [45]). Pomel [23] did not propose any precise taxonomic affinities for *Sauromorus*, and only commented about shared features but also differences with the extant *Lacerta* Linnaeus, 1758 [46]. Subsequently, *Sauromorus* was regarded as a scincid [47, 48], while the taxon was later treated as an anguid [49, 50]. Hoffstetter [51] mentioned that the type material of *Sauromorus ambiguus* and *S. lacertinus* was lost and, moreover, as it had never been figured, he suggested that these taxa are of uncertain affinities, and treated them as nomina nuda. However, being nomina nuda cannot be the case, as Pomel [23] provided a brief description for the taxa, so the names *Sauromorus ambiguus* and *S. lacertinus* have to be considered available for nomenclature purposes according to ICZN [52] as they both fulfil the minimum requirements for availability of zoological names published before 1931. As such, *Sauromorus ambiguus* and *S. lacertinus* are nomina dubia, as it has already been previously suggested [45, 53, 54]. This being said, both *Sauromorus* spp. can only be tentatively identified as indeterminate lizards.

For the third squamate species from Langy, *Ophidion antiquus*, only a brief description of a parietal was provided [23], again without any accompanying figure, and also a report of the presence of vertebrae that could be referred to the same taxon. A brief mention of this species, but without any further description, also appeared shortly after [55]. Rochebrune [56], on the other hand, regarded *O. antiquus* to be an amphisbaenian instead of a snake, but Hoffstetter [57] returned it back to snakes, stating that the morphology of the parietal was indeed reminiscent of booids. Kuhn [58] eventually found out that the original genus name *Ophidion* was in fact a junior homonym of the fish *Ophidion* Linnaeus, 1758 [46], and therefore a new genus name was created, *Ophidioniscus* Kuhn, 1963 [58], in order to accommodate the species from Langy. Hoffstetter and Rage [3] continued to regard that the described parietal hints at booid affinities, and particularly to “erycines”, and they further tentatively envisaged that it could even eventually pertain to the “erycine” *Bransateryx*, which was already documented from the area of Saint-Gérand-le-Puy. Nevertheless, in the same paper, *O. antiquus* was treated as a nomen nudum [3], which, however, cannot be the case, as (similarly to *Sauromorus* discussed above) Pomel [23] had provided a minimum description and therefore, this action had rendered the name available. Indeed, Rage [59] clarified that the name is available for nomenclatural purposes, but nevertheless, considered it as a nomen dubium, with the material representing an indeterminate booid. It was further suggested that the vertebrae were not part of the type series and that only the, now lost, parietal was the holotype [59], an opinion shared by others [8], who treated the vertebrae as referred material. Regardless the case, the material has never been figured and not enough can be gleaned from the original generalized description [23]. *Ophidion antiquus* is regarded to be a nomen dubium and the material can be identified probably solely as Constrictores indet.

Few decades later, Lydekker [24] documented squamates from Saint-Gérand-le-Puy, referring a presacral and a caudal lizard vertebrae to *Placosaurus margariticeps* and some snake trunk vertebrae to *Paleryx rhombifer* and *Paleryx depressus*. Besides the fact that all these three species had original stratigraphic distributions that were significantly older (i.e., Eocene) than the early Miocene age of the French locality, more recent revisions of Lydekker’s [24] material have since referred that material to different taxa. As such, Lydekker’s [24] referral to *Placosaurus margariticeps* (a large lizard from Quercy, originally described as *Varanus margariticeps* Gervais, 1876 [60] (see [43, 61]), should be better reidentified simply as an indeterminate anguid. As for Lydekker’s [24] snakes, *Paleryx rhombifer* Owen, 1850 [62], and its currently considered junior synonym *Paleryx depressus* Owen, 1850, are regarded to pertain to Constrictores and their known distribution is confined to the late Eocene of England (see [63]). As such, the purported occurrence of *Paleryx* in Saint-Gérand-le-Puy has been reinterpreted as probably pertaining to *Bransateryx* [63, 64].

Important squamate discoveries from Saint-Gérand-le-Puy took place also in the twentieth century. As such, a new gekkotan genus and species was established on the basis of cranial material from Saint-Gérand-le-Puy, *Gerandogekko arambourgi* Hoffstetter, 1946 [2]. In addition, snake cranial and trunk vertebral material from Saint-Gérand-le-Puy was eventually described and referred to *Bransateryx* [3].

The presence of a viperid in Saint-Gérand-le-Puy was highlighted by Szyndlar and Rage [27, 65], who cited that it was initially mentioned by Hoffstetter[66]. However, the latter author indeed mentioned a Miocene French viperid but had made no explicit mention of a locality, and in fact, he only mentioned that it originated from the “Aquitanien de la Limagne” ([66], p. 659). Subsequently, viperid material from Saint-Gérand-le-Puy was described and figured [27], in which it was referred to the *Vipera aspis* complex. Furthermore, Szyndlar and Rage [65] considered that an original mention of a maxilla from the French Aquitanian made by Hoffstetter [67] could probably pertain to a specimen from Saint-Gérand-le-Puy, but that specimen was, unfortunately, not ultimately located [65]. Because of its age, the Saint-Gérand-le-Puy viperid has been considered to be of high importance, being treated as one of the oldest known viperids, and in particular the oldest known member of Viperinae [27, 68].

The nearby localities within the Saint-Gérand-le-Puy complex have also yielded squamates. From Poncenat (MN 2a), a new lizard species, *Lacerta poncenatensis* Müller, 1996 was established, which was only referred to *Lacerta* sensu lato [25]. Again from Poncenat, the same author described cranial remains of *Bransateryx* and indeterminate snakes [26]. From the same site as well, the presence of the Oligocene species *Lacerta filholi* Augé, 1988 [69] was reported [61], however, no figure, description or even collection number of that material was provided. From Chavroches (MN 2), there is only a non-documented mention of *Bransateryx vireti* [8]. The fossil-rich site of Montaigu-le-Blin (MN 2), the reference locality of the MN 2 age, is the type locality of another gekkotan, the sphaerodactylid *Euleptes gallica* Müller, 2001 [6]. From the same site, there are also reports of an indeterminate “anilioid” snake and *Bransateryx vireti* [8], however, these are both undocumented. Finally, from the older, nearby locality of Gannat (MP 30–MN 1), cranial material of the lacertid *Pseudeumeces cadurcensis* (Filhol, 1877) [55] was described [70].

## Methods

All material described herein is permanently curated at the collections of PIMUZ. Specimens from Saint-Gérand-le-Puy were acquired by PIMUZ during 1998, while two other specimens from the nearby sites of Montaigu-le-Blin and Gannat were collected during 1984 by Hugo Bucher (PIMUZ). A left dentary (PIMUZ A/III 4656) and two parietals (PIMUZ A/III 4626 and PIMUZ A/III 4627) were micro-CT scanned using a Nikon XTH 225 ST CT Scanner housed at the Anthropological Department of the University of Zurich. The micro-computed tomography scan of the specimen PIMUZ A/III 4656 was taken with a voltage of 124 kV and a current of 182 µA, yielding a voxel size of 0.00722 mm, PIMUZ A/III 4626 with 127 kV and 185 µA yielding a voxel size of 0.00761 mm, and PIMUZ A/III 4627 with 127 kV and 170 µA yielding a voxel size of 0.00757 mm. In all three scans, a 1 mm copper filter was used. The datasets were then visualized using Materialise Mimics Version 23. The micro-CT scan data and the 3D surface files (.PLYs) are available on Morphosource repository (https://www.morphosource.org/) (see “Availability of data and materials” for details).

### Geological and palaeoecological settings

The fossiliferous area of Saint-Gérand-le-Puy (the “Saint-Gérand-le-Puy complex”) is situated near the homonymous town at the French Massif Central, at the department of Allier, in the region of Auvergne-Rhône-Alpes, France. It consists of several quarries near the towns of Saint-Gérand-le-Puy, Montaigu-le-Blin, and Boucé, and, in a more extended sense, also others near the towns of Bransat, Chavroches, Langy, and Saulcet. All faunas have been considered to be more or less contemporary [71] and in any case, the different quarries lie within the Aquitanian (MN 1–2, mainly at MN 2). The most fossil rich quarries are Montaigu-le-Blin (or Montaigu) (MN 2a), Langy (MP 30 or MN 2), Chavroches (MN 2a), Saulcet (MN 1⁄ 2a), Poncenat (MN 2), Cluzel (MN 2a), Billy (Billy-Créchy) (upper level, MN 1), Chavroches (MN 2a), Gondailly, and Mont Merle (MN 2a) [71–76]. Notably, among them, Montaigu-le-Blin is the reference locality of the European Mammal Neogene Unit MN 2 [77]. Fossils are found in limestones, known in the literature under the names “Calcaire à Phryganes”, “Calcaires a Indusies” (= Indusial Limestone), “calcaires en chou-fleur”, and Phryganea Limestones. Certain latest Oligocene sites, such as Gannat and Coderet, are also in the vicinity. The different quarries of Gannat are older, considered to be within the range of MP 30–MN 1 [73]—for reasons of convenience and completeness, Gannat is included in this study. More details about the geology of the area can be found elsewhere [71, 78–80].

The limestone deposits of the Saint-Gérand-le-Puy complex are already known since the beginning of the nineteenth century [81, 82], with subsequent vertebrate fossil descriptions taking place in the following decades [83–85]. However, as much of the recovered fossils was simply the byproduct of limestone exploitation, most of the nineteenth century faunal assemblages are in fact a mix of specimens originating from different quarries within the area [74]; such cases with mixed fossil assemblages within areas of resources exploitations are not uncommon in palaeontological collections of nineteenth century Europe, an example being the famous Phosphorites du Quercy [43, 86, 87]. In the old literature, although at certain works detailed quarry information do exist (e.g., [23]), in most cases, such data are lacking and the only available information is simply “Saint-Gérand-le-Puy” (e.g., [24, 88]). Such is the case with the PIMUZ material, as all specimens bear only this general information, with the exception of the lacertid dentary that was collected from Montaigu-le-Blin and a snake vertebra from Gannat. Even if not strictly sympatric or contemporary, the age of all these squamates described herein is earliest Miocene (MN 1–2, mainly c. MN 2), while the single vertebra from Gannat is slightly older (MP 30–MN 1).

Recent palaeoenvironmental reconstuctions have suggested that during the early Miocene, the area of Saint-Gérand-le-Puy was located at the margins of a large lake approximately 70 km in length) [74, 89]. Both terrestrial and freshwater aquatic (fluvial/lacustrine) taxa have been identified in the different quarries of the Saint-Gérand-le-Puy complex [75]. Apart from squamates, other reptiles include turtles and crocodylians [24, 84, 88, 90–94]. Besides these reptiles, Saint-Gérand-le-Puy is well known for its rich and diverse assemblage of mammals [74, 85, 95] and birds [72, 75, 76, 79, 96–98], as well as remains of amphibians, insects, and mollusks [80].

## Results

### Systematic Palaeontology

Squamata Oppel, 1811 [99]

Lacertidae Oppel, 1811 [99]

*Janosikia* Čerňanský, Klembara, and Smith, 2016 [15]

*Janosikia* sp.

**Fig. 1:**
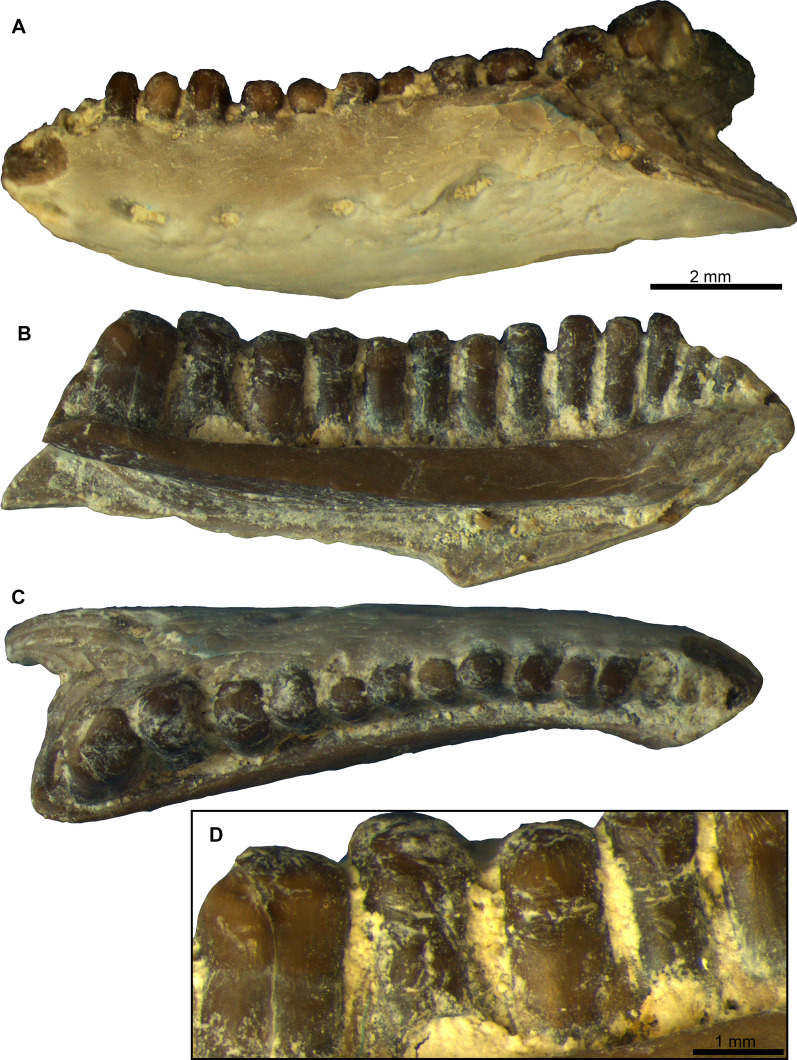
*Janosikia* sp. **A–C**, Left dentary PIMUZ A/III 4656 in **A**, labial, **B**, medial, and **C**, dorsal views; **D**, close up of the posterior teeth of the same specimen in medial view, showing the striation

**Fig. 2:**
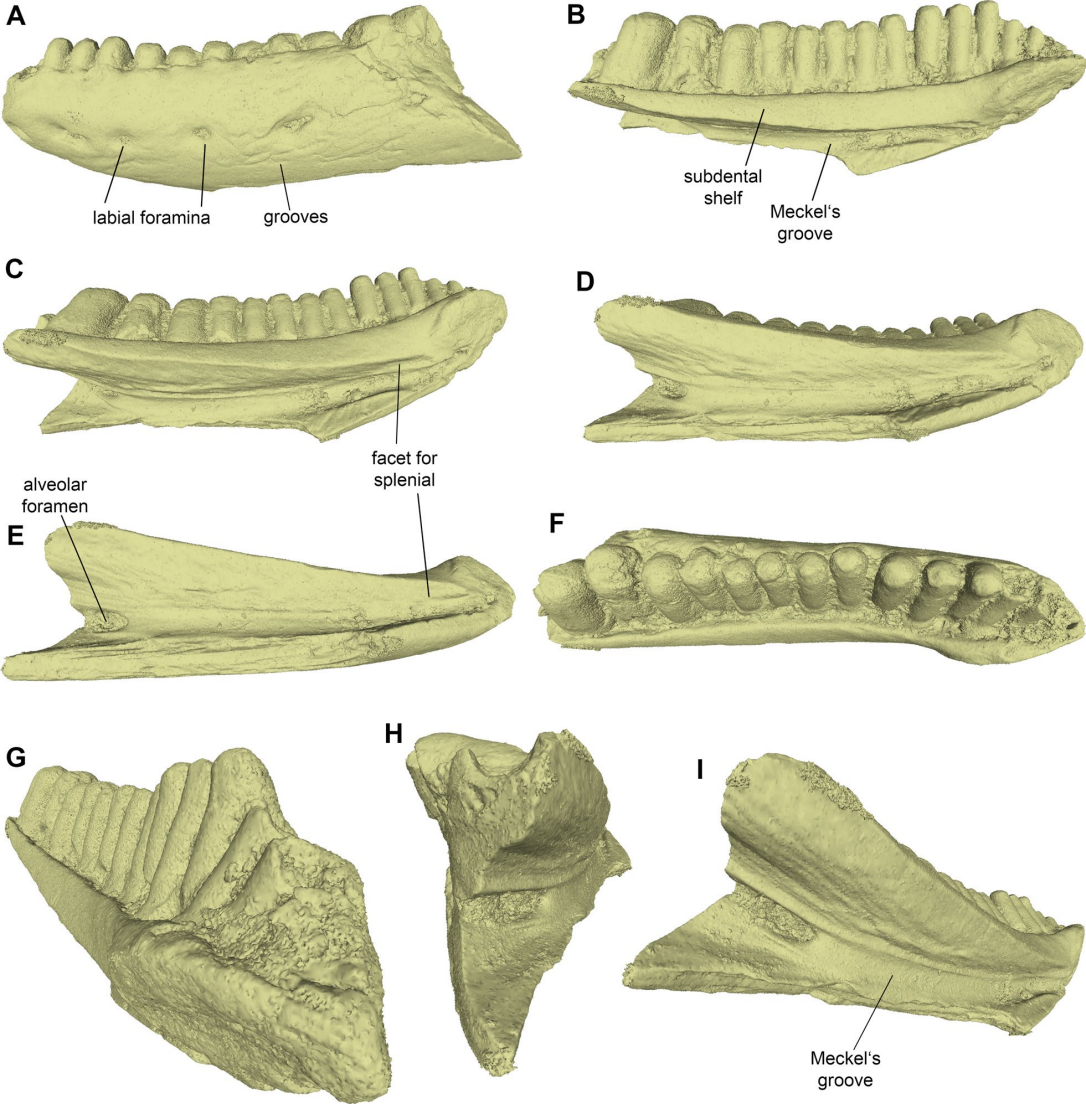
3D models of the left dentary PIMUZ A/III 4656 of *Janosikia* sp. in **A** labial, **B** medial, **C–D** ventromedial, **E** ventral, **F** dorsal, **G** anterodorsal, **H** posterior, and **I** posteroventral views

**Fig. 3:**
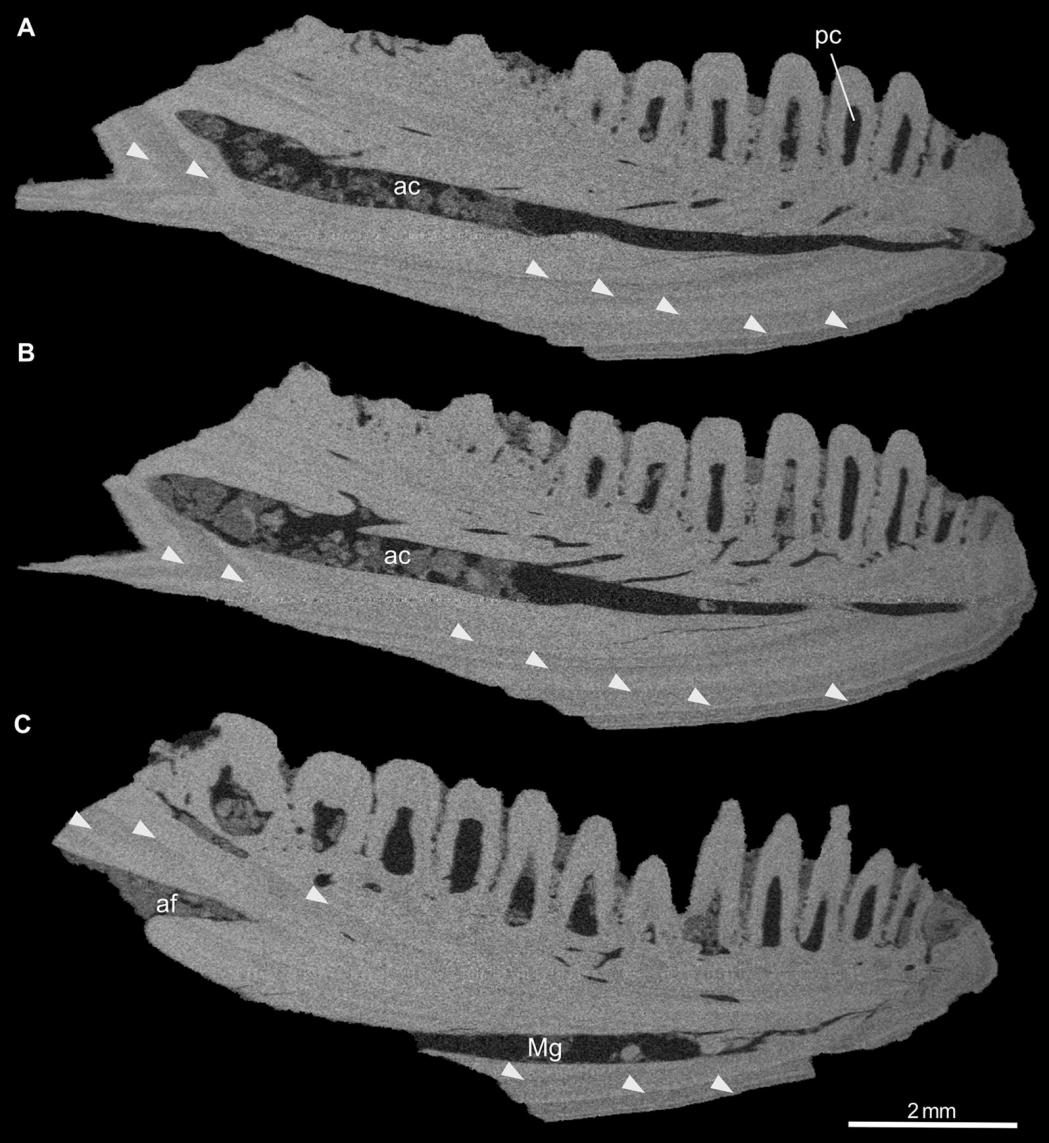
Virtual parasagittal sections through the left dentary PIMUZ A/III 4656 of *Janosikia* sp. The image sequence **A** to **C**: slices from lateral to medial. The white arrowheads indicate growth marks in the dentary bone. Abbreviations: ac, alveolar canal; af, alveolar foramen; Mg, Meckel’s groove; pc, pulp cavity

Figures 1, 2, 3, Additional File 1 and Additional File 2.

**Material**: *Montaigu-le-Blin*: a left dentary (PIMUZ A/III 4656).

**Description**: The left dentary PIMUZ A/III 4656 is incomplete, missing parts of its posterior, anteriormost, and ventral portions (Figs. 1, 2). It is slightly ventrally arched, while its posterodorsal portion is slightly inclined. The alveolar crest preserves 13 (partial or complete) teeth, but there are at least two other empty tooth positions. In medial view (Figs. 1B, 2B), the Meckel’s groove is deep and fully open; it is wider in the posterior and middle portions of the dentary but its width gradually diminishes towards its anterior edge. The symphyseal region is eroded. The subdental shelf is straight to slightly concave; it is thick in its anterior and mid-portions, being much narrower at its posterior part. Its anterior portion is not markedly elevated relative to the posterior portion of the shelf. A facet for the splenial is present in its ventral side. The alveolar foramen is relatively large, situated approximately at the level of the posteriormost preserved tooth. In labial view (Figs. 1A, 2A), the surface of the dentary is roughly smooth, alternated, however, with an array of irregular grooves and distinct ridges, which are most prominent at its mid-height and ventral portions. The labial surface of the dentary is pierced by at least five large labial foramina. Dentition is pleurodont and strongly heterodont, being amblyodont at the posterior portion of the dentary. Teeth are closely spaced. They gradually increase in size and robustness posteriorly. As such, teeth at the anterior portion of the dentary are small and slender, while the posteriormost ones preserved are considerably robust, forming blunt cylinders. The tooth crowns of all teeth bear distinct striations (Fig. 1D). Such tooth striation is more prominent at the medial side of the teeth. No clear accessory cusps are present on the preserved teeth.

**Virtual microanatomy and histology:** The micro-CT scan of the dentary PIMUZ A/III 4656 revealed a few internal structures, including the partially sediment-filled alveolar canal, the pulp cavities of each tooth, a fine branching neuro-vascular network of thin channels connecting the alveolar canal either with the pulp cavities or with foramina opening on the lateral bone surface, as well as a set of growth marks in the dentary bone (Fig. 3). The growth marks appear as brighter and darker colored triangular cones (different grey tones reflecting slightly different densities in the bone matrix and thus different growth periods; see [100]) at the posterior margin of the dentary and as gently curved parallel lines in the anterior part (Fig. 3). Seven growth marks could be counted in the dentary.

**Comments**: The dentary PIMUZ A/III 4656 can be referred to the lacertid genus *Janosikia* on the basis of its relatively low tooth count (around 15 preserved tooth positions), closely spaced teeth that are more slender anteriorly and become more robust posteriorly, presence of striation on the teeth, convex ventral margin of the bone, and the anterior, symphyseal region of the subdental shelf being not markedly elevated relative to the posterior portion of the shelf (see [15]). A more precise determination, i.e., whether it pertains to the type species, *Janosikia ulmensis* (Gerhardt, 1903) [12] from the early Miocene of southern Germany [15] or some different congeneric form, cannot be made as the dentary from Montaigu-le-Blin is incomplete. It therefore does not preserve important diagnostic features in its posterior portion, for example, in the coronoid and the shape/size of the posteriormost teeth. The distinct grooves and ridges present in the labial surface of the dentary from Montaigu-le-Blin seem to be rather prominent compared with other previously published specimens of *Janosikia*, where that surface appears to be smoother. Whether, however, this distinctly grooved pattern observed in the Montaigu-le-Blin specimen bears some taxonomic utility or is simply attributed to some taphonomical or preservational reasons, cannot be further evaluated.

Anguimorpha Fürbringer, 1900 [101]

Anguidae Gray, 1825 [102]

Anguinae Gray, 1825 [102]

*Ophisaurus* Daudin, 1803 [103]

*Ophisaurus holeci* Klembara, 2015 [104]

**Fig. 4:**
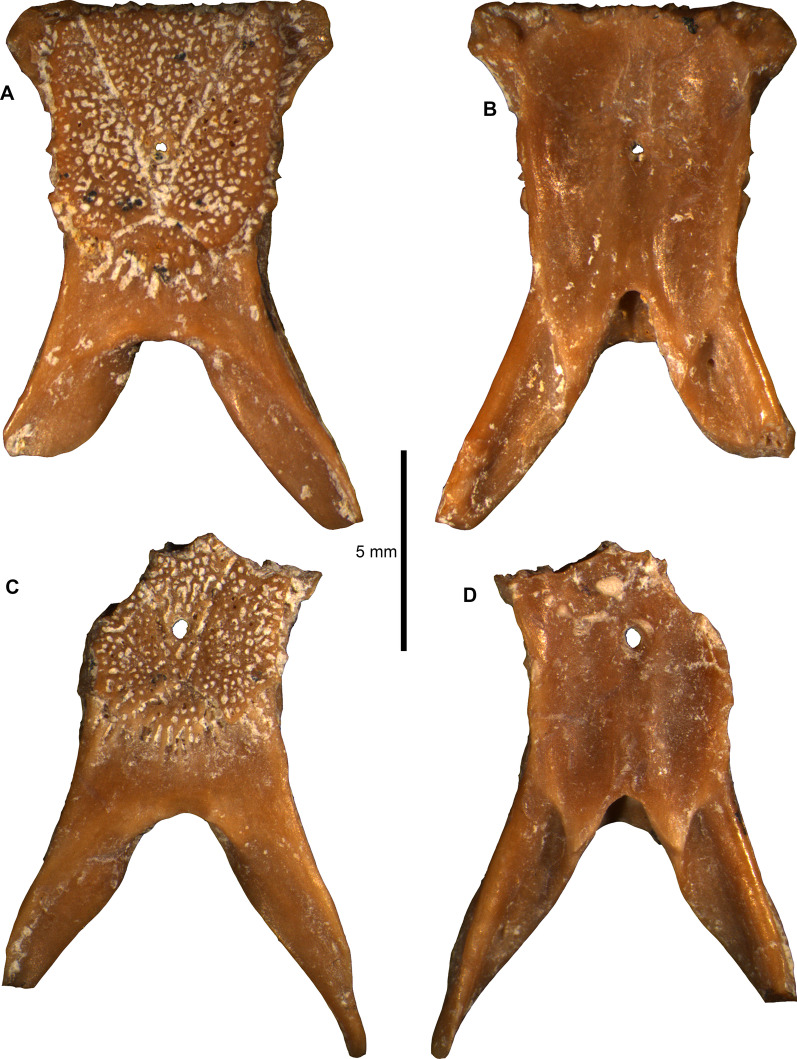
*Ophisaurus holeci* parietals. **A–B** parietal PIMUZ A/III 4626 in **A** dorsal and **B** ventral views; **C–D** parietal PIMUZ A/III 4627 in **C** dorsal, and **D** ventral views

**Fig. 5:**
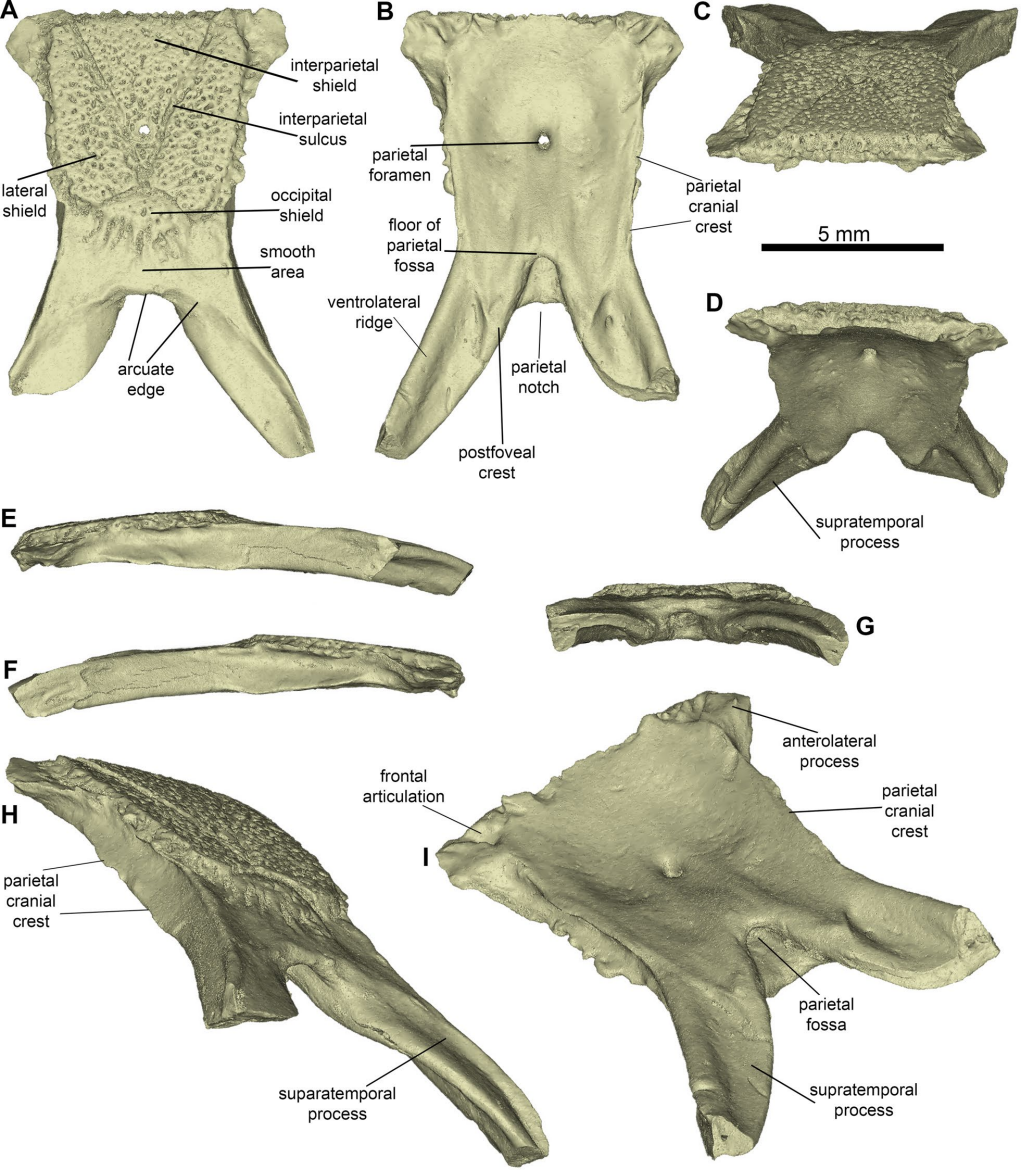
3D models of the parietal PIMUZ A/III 4626 of *Ophisaurus holeci* in **A** dorsal, **B** ventral, **C** anterodorsal, **D** anteroventral, **E** left lateral, **F** right lateral, **G** posterior, **H** posterolateral, and **I** posteroventral views

**Fig. 6:**
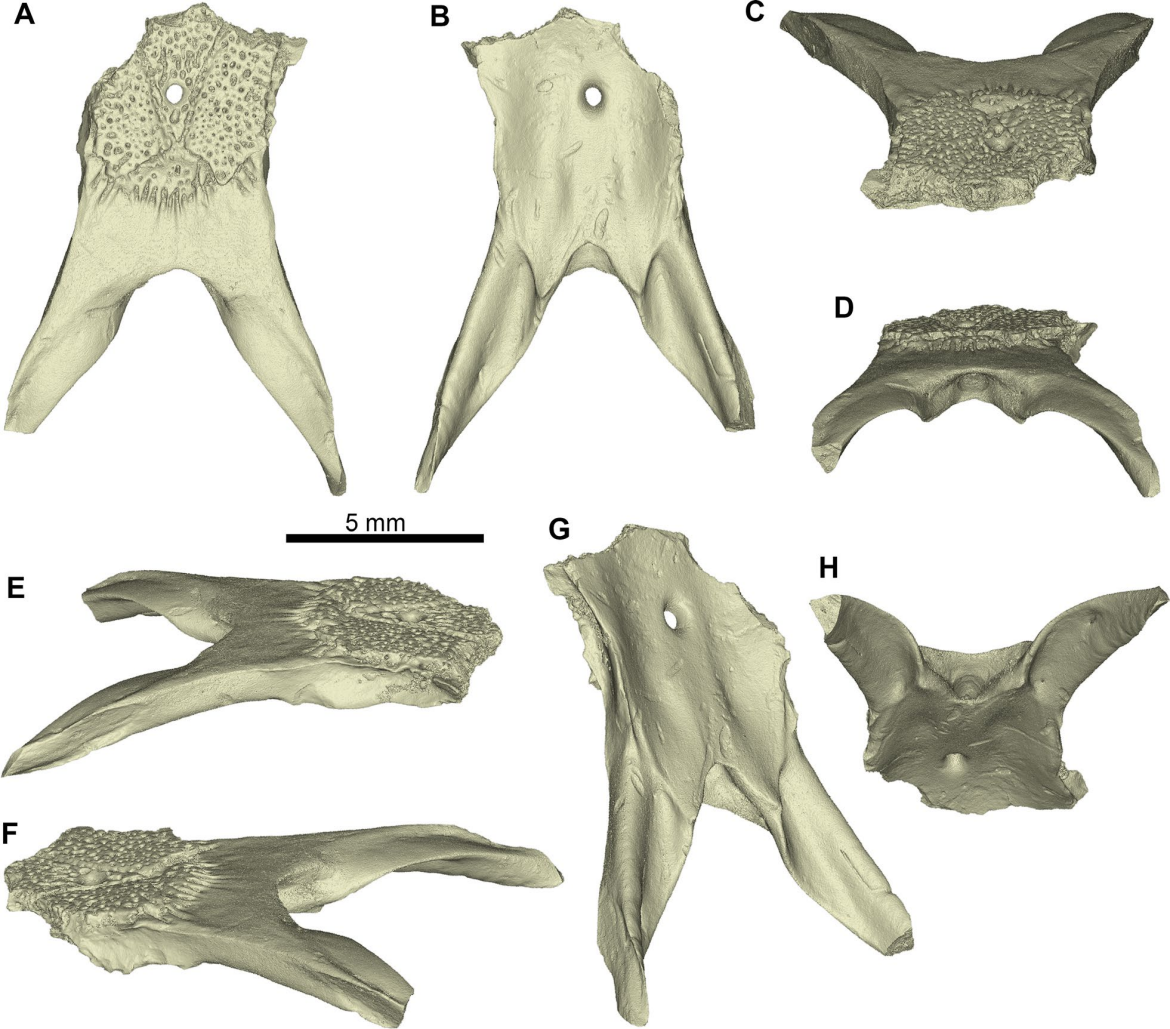
3D models of the parietal PIMUZ A/III 4627 of *Ophisaurus holeci* in **A** dorsal, **B** ventral, **C** anterodorsal, **D** posterodorsal, **E** right dorsolateral, **F** left dorsolateral, **G** ventrolateral, and **H** posteroventral views

**Fig. 7:**
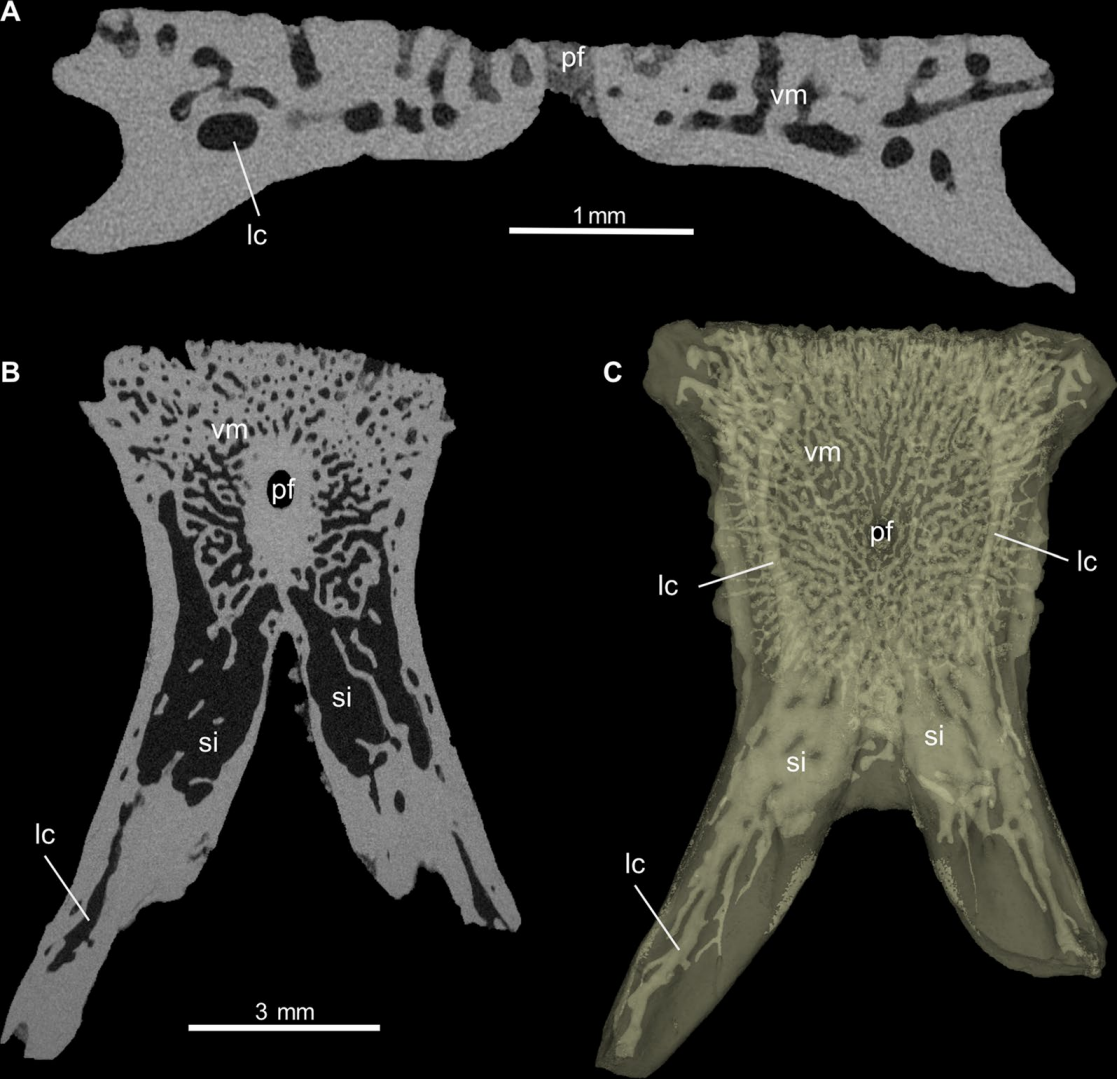
Virtual sections and transparent 3D model of parietal (PIMUZ A/III 4626) of *Ophisaurus holeci* in **A** coronal section, **B** axial section, and **C** 3D model in ventral view. Note that the mesh of fine radiating and branching vascular channels is restricted to the dorsal portion of the parietal table, while the larger vessels and sinuses/cavities lie more ventrally. *lc* larger canal; *pf* parietal foramen; *si* sinus; *vm* vascular mesh

Figures 4, 5, 6, 7, Additional Files 3, 4, 5, 6.

**Material**: *Saint-Gérand-le-Puy*: two parietals (PIMUZ A/III 4626 and PIMUZ A/III 4627).

**Description**: The two parietals are relatively well preserved. PIMUZ A/III 4626 is the most complete, lacking only small part of the left supratemporal process (Figs. 4A–B, 5). PIMUZ A/III 4627 is more incomplete, missing its anteriormost edge and the posteriormost tip of the left supratemporal process (Figs. 4C–D, 6). Their morphology is generally similar; however, PIMUZ A/III 4627 is relatively slenderer. The description is based on both specimens.

In dorsal view (Figs. 4A, C, 5A, 6A), the parietal table is covered by a prominent sculptured surface. The sculptured surface is slightly longer than wide (at the level of its mid-length and mid-width). The lateral margins of the sculptured surface almost coincide with the lateral margins of the parietal table. The ornamentation consists of distinct ridges and deep grooves and pits. The sculptured surface, which is divided by distinct sulci (i.e., interparietal and occipital sulci) into an interparietal shield, an occipital shield, and two lateral shields. The anterior end of the interparietal sulcus lies medial to the anterolateral corner of the sculptured surface. The parietal foramen is located at the posterior portion of the interparietal shield—it is proportionately larger in PIMUZ A/III 4627. The sulcus in the junction of the interparietal and occipital shields is extremely tiny and almost incipient. The occipital shield is triangular to slightly rhomboidal, as its posterior margin morphology is relatively concave. The anterolateral processes are preserved in PIMUZ A/III 4626, where they are well developed. The smooth area of the parietal table is larger in PIMUZ A/III 4627; in both specimens, its anteroposterior length in medial plane is larger than the anteroposterior length of the occipital shield. The supratemporal processes are almost straight. The arch-like arcuate edge is on the dorsal surface of the anterior halves of the supratemporal processes; it is straighter in PIMUZ A/III 4626, while it is more convex in PIMUZ A/III 4627.

In ventral view (Figs. 4B, D, 5B, 6B), the frontal tab is prominent in PIMUZ A/III 4626 (not preserved in PIMUZ A/III 4627). There is no muscular surface, as seen in species of *Pseudopus* Merrem, 1820 [105]. The parietal cranial crest lies almost at the level of the lateral margin of the parietal table. The parietal cranial crest is high and sharp especially in its mid-length, but becomes rather low in its anterior and posterior portions; the posterior portion of the parietal crest is lowest at its junction with the ventrolateral ridge of the supratemporal process. The postfoveal crest is short anteroposteriorly and low dorsoventrally—it is more prominent in PIMUZ A/III 4627. The supratemporal processes are straight—they mostly diverge posteriorly in PIMUZ A/III 4626 but not so in PIMUZ A/III 4627. The anterior end of the supratemporal process joins the posterior section of the parietal cranial crest at or slightly posterior to the posteromedian margin of the parietal fossa floor. The ventrolateral ridge of the supratemporal process is robust and coincides with the process’s lateral margin anterior to the supratemporal articulation.

**Virtual microanatomy and histology:** The two micro-CT scans of the parietals revealed a robust bone structure with a very similar internal microanatomy in terms of an extensive vascular network, whereas finer histological details (growth marks, cell lacunae, etc.) of the bone were not visible. Both parietals have the most extensive interconnecting vascular spaces just beneath the smooth area posterior to the parietal table (Fig. 7, Additional Files 4, 6). From here, a network of thinner interconnected channels extends posterolaterally into each supratemporal process, while two main vessels extend anterolaterally towards the anterior bone margin. These two main vessels connect dorsally with a fine regular meshwork of thin channels that perforate the ornamented parietal table and open up into numerous small dorsal foramina on the sculptured bone surface.

**Comments**: The two parietals from Saint-Gérand-le-Puy can be assigned to *Ophisaurus holeci* on the basis of the following diagnostic features: (i) the anterior end of the interparietal sulcus lies medial to the anterolateral corner of the sculptured surface; (ii) the anterior end of the ventrolateral ridge of the supratemporal process joins the parietal cranial crest at or slightly posterior to the posteromedian margin of the floor of the parietal fossa; (iii) the posterior portion of the parietal cranial crest is rather low, particularly at its junction with the ventrolateral ridge of the supratemporal process; (iv) the supratemporal process is straight; (v) the base of the supratemporal process is mediolaterally narrow; (vi) the presence of a short postfoveal crest; (vii) a long anterior section of the parietal cranial crest is more or less distinctly concave (though this latter feature is not so prominent in the Saint-Gérand-le-Puy parietals) (features from [104, 106]). Differences among the French material and the holotype and previously referred parietals can be attributed to intraspecific variability. Accordingly, differences between the two parietals from Saint-Gérand-le-Puy can be attributed to intraspecific or ontogenetic variation, with the slenderer specimen (PIMUZ A/III 4627) pertaining probably to an earlier ontogenetic stage.

It is further worth noting that another anguine species has been previously described from the vicinity of Saint-Gérand-le-Puy. This is *Ophisauromimus coderetensis* (Augé, 2005) [61] from the latest Oligocene (MP 30) of Coderet, only a few (around 20) km away from Saint-Gérand-le-Puy [61]. This taxon was originally described as a species of the extant Asian genus *Dopasia* Gray, 1853 [107], by Augé [61], until it was eventually placed in its own genus, *Ophisauromimus* Čerňanský, Klembara, and Müller, 2016, by [108]. *Ophisauromimus coderetensis* was typified by a dentary from Coderet, with an additional fragmentary parietal from that locality referred to the same species [61]; other cranial remains (but not a parietal) have been referred to this species from the early Oligocene of France and the late Oligocene of Germany [61, 108]. This single described parietal from Coderet is too fragmentary to afford any reliable comparison with the new parietals from Saint-Gérand-le-Puy. In any case, although the dentary morphology of *Ophisaurus holeci* is not known with certainty, the dentary of *Ophisauromimus coderetensis* exhibits considerable differences with that of *Ophisaurus* spp. [108]. Therefore, we can exclude affinities of the Saint-Gérand-le-Puy parietals with *Ophisauromimus coderetensis*.

*Ophisaurus* sp.

Figures 8 and 9

**Fig. 8:**
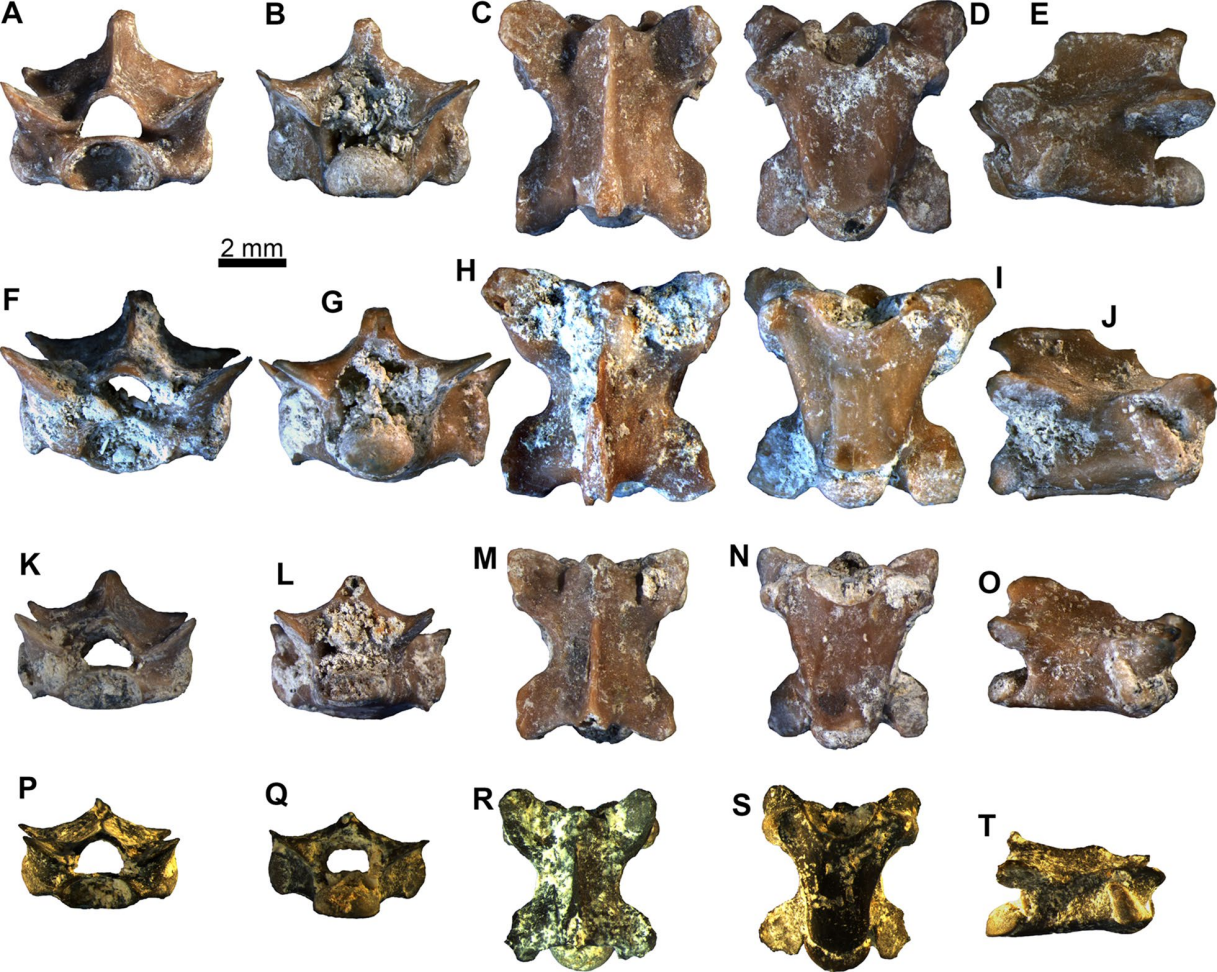
*Ophisaurus* sp. presacral vertebrae: **A–E** presacral vertebra PIMUZ A/III 4655 in **A** anterior, **B** posterior, **C** dorsal, **D** ventral, and **E** left lateral views; **F–J** presacral vertebra PIMUZ A/III 4637 in **F** anterior, **G** posterior, **H** dorsal, **I** ventral, and **J** right lateral views; **K–O** presacral vertebra PIMUZ A/III 4638 in **K** anterior, **L** posterior, **M** dorsal, **N** ventral, and **O** right lateral views; **P–T** presacral vertebra PIMUZ A/III 4639 in **P** anterior, **Q** posterior, **R**, dorsal, **S** ventral, and **T** right lateral views

**Fig. 9:**
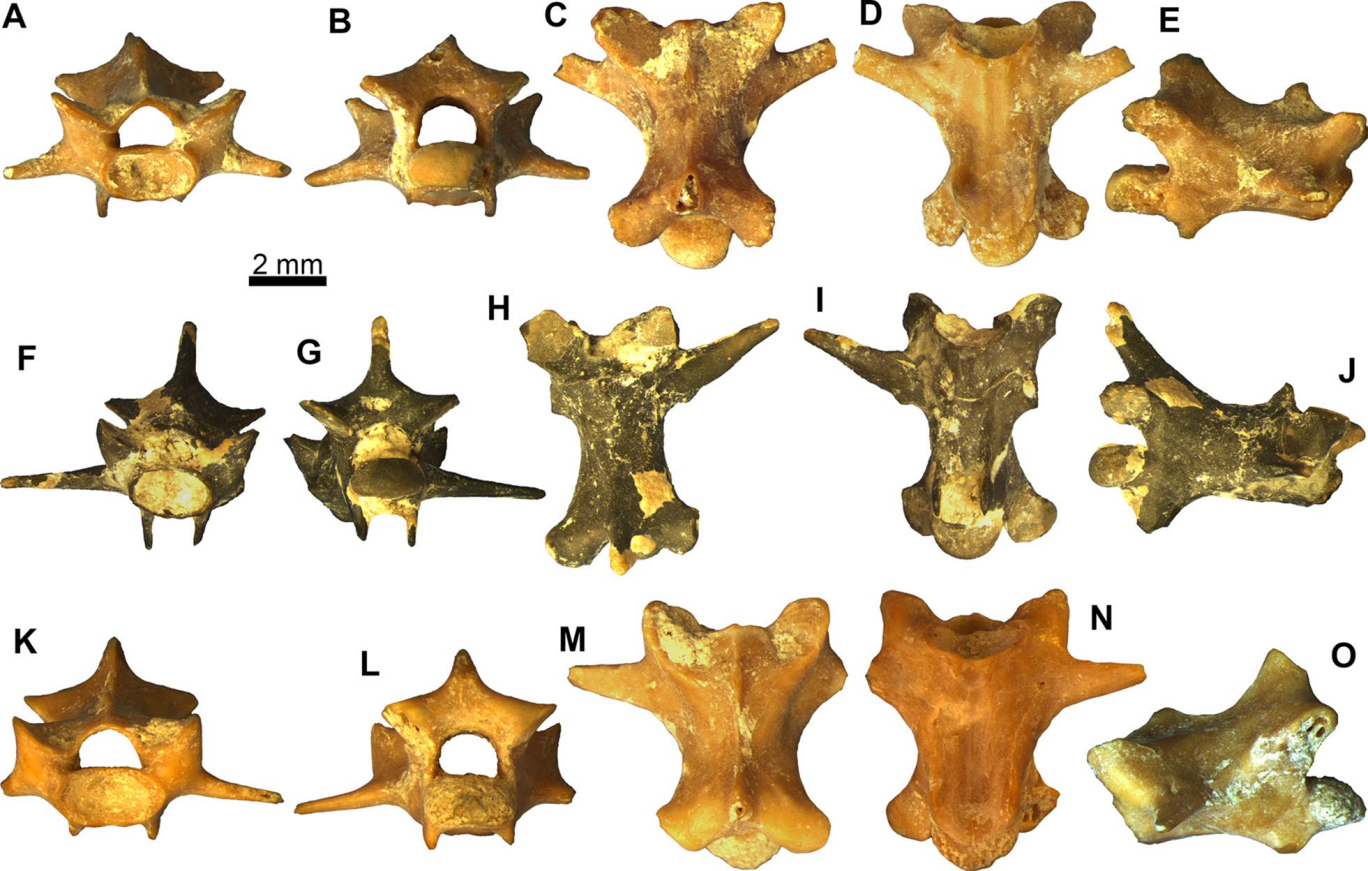
*Ophisaurus* sp. caudal vertebrae: **A–E** caudal vertebra PIMUZ A/III 4640 in **A** anterior, **B** posterior, **C** dorsal, **D** ventral, and **E** right lateral views; **F–J** caudal vertebra PIMUZ A/III 4645 in **F** anterior, **G** posterior, **H** dorsal, **I** ventral, and **J** right lateral views; **K–O** caudal vertebra PIMUZ A/III 4646 in **K** anterior, **L** posterior, **M** dorsal, **N** ventral, and **O** left lateral views

**Material**: *Saint-Gérand-le-Puy*: ten presacral vertebrae (PIMUZ A/III 4637-PIMUZ A/III 4639, PIMUZ A/III 4641-PIMUZ A/III 4644, PIMUZ A/III 4655, PIMUZ A/III 4657, and PIMUZ A/III 4680) and ten caudal vertebrae (PIMUZ A/III 4640, PIMUZ A/III 4645-PIMUZ A/III 4652, and PIMUZ A/III 4658).

**Description**: The presacral vertebrae have centrum lengths ranging between 4.0 and 5.7 mm (Fig. 8). They are relatively dorsoventrally compressed. PIMUZ A/III 4657 is the only cervical vertebra—it bears a hypapophysis, however, this is rather damaged and therefore its shape and extent cannot be evaluated with certainty. All the rest of the presacral vertebrae lack hypapophyses, clearly therefore pertaining to the post-cervical region of the column. The ventral surface of the centrum in these vertebrae is relatively flat, occasionally with few subcentral foramina. There is no precondylar constriction. The lateral margins of the centrum are relatively concave (e.g., Fig. 8N, S), although in the largest vertebrae these appear to be more or less straight (Fig. 8D, I). The prezygapophyses are dorsally inclined in anterior view. The neural spine is incomplete in most specimens. Nevertheless, it is almost fully preserved in PIMUZ A/III 4655, where it is trapezoidal, being dorsoventrally low, anteroposteriorly long, and slightly posteriorly inclined (Fig. 8E). The neural spine is thicker in dorsal view at the posterior portion of the neural arch. The neural canal is large, with its height being larger to that of the cotyle (especially in PIMUZ A/III 4638 and PIMUZ A/III 4639; Fig. 8K, P); nevertheless, in the two largest specimens, PIMUZ A/III 4655 and PIMUZ A/III 4637, the height of the neural canal is smaller than that of the cotyle (Fig. 8A, F). The cotyle and condyle are markedly depressed. The synapophyses are elongated.

The caudal vertebrae have centrum lengths ranging between 4.3 and 6.1 mm (Fig. 9). They are anteroposteriorly elongated and considerably narrow. In the most gracile of them (PIMUZ A/III 4651), this elongation is at its most extreme. In most of them, the transverse processes and haemal arches are broken, however, in few specimens, the former structures are complete (e.g., PIMUZ A/III 4648). The neural spine is also usually incomplete—when complete, it can be dorsoventrally high and anteroposteriorly thin; it is practically confined solely to the posteriormost portion of the neural arch and is inclined posteriorly (e.g., PIMUZ A/III 4648). The prezygapophyses and postzygapophyses are relatively small. The former are dorsally inclined in anterior view. Cotyle and condyle are rather depressed. The transverse processes are anteroventrally oriented—they are large and their bases are located in the anterior portion of the centrum. The chevron bones forming haemal arches are fused to the centrum, located at the posterior portion of the vertebra, relatively near the condyle. An autotomic plane can be present, being more visible in PIMUZ A/III 4645 (Fig. 9I).

**Remarks**: The presacral vertebrae can be referred to *Ophisaurus* on the basis of the lateral margins (subcentral ridges) of the centrum being concave instead of straight, the height of the neural canal being larger to that of the cotyle, and the neural spine being trapezoidal with the length of its basal portion being bigger than its height (characters from [109]). We nevertheless, have to highlight here that two among the largest vertebrae (PIMUZ A/III 4655 and PIMUZ A/III 4637) deviate from this typical *Ophisaurus* morphology, in respect to their neural canal height being smaller than that of the cotyle and the lateral margins of the centrum being more or less straight and not so concave. In fact, in these two latter features, PIMUZ A/III 4655 and PIMUZ A/III 4637 are more reminiscent of *Pseudopus apodus* (Pallas, 1775) [110] instead of *Ophisaurus* (characters from [109]); however, the shape of the neural spine of these two vertebrae is rather different from that of extant *P. apodus*. That being said, we would here refrain from considering these two largest vertebrae as pertaining to *Pseudopus* and instead regard them as being congeneric with the rest of the anguine material from Saint-Gérand-le-Puy. If our interpretation is correct, then these two vertebral features (i.e., the ratio of neural canal height to cotyle height and the concavity of the lateral margins of the centrum) may have been relatively more variable among earliest Miocene taxa of *Ophisaurus* and *Pseudopus*. More abundant anguine vertebral material from the locality may eventually confirm or refute this suggestion.

As for the caudal vertebrae, they display typical anguine features, i.e., the fused haemal arches and the presence of an autotomic plane (absent in *Pseudopus*). We tentatively refer them as well to *Ophisaurus* by the presence of an autotomic plane (at least in certain specimens) and the overall shape [109].

Although it remains possible that this vertebral material pertains to the same species as the above described parietals (i.e., *Ophisaurus holeci*), a more precise determination is not possible on the absence of species-level diagnostic features, as well as the fact that *Ophisaurus* was rather diverse during the early Miocene of Europe, with more than one congeneric species co-occurring in certain localities (e.g., [104, 106]). It is worth noting that so far published vertebrae which have been reliably referred to *O. holeci* based on articulated skeletons, show a typical *Ophisaurus*-like vertebral morphology [111]. On the other hand though, a recent phylogenetic analysis has recovered *O. holeci* as most closely related to the genus *Ophisauriscus* Kuhn, 1940 [13], from the Eocene of Germany instead of other *Ophisaurus* spp. [14]. Nevertheless, despite the fact that *Ophisauriscus* is known from several rather complete articulated skeletons from the Eocene fossil Lagerstätten localities of Geiseltal and Messel (see [13, 112]), certain of its vertebral features are not as yet adequately known.

Serpentes Linnaeus, 1758 [46]

Constrictores Oppel, 1811 [113] (sensu [114]).

“erycines” (sensu [115]).

*Bransateryx* Hoffstetter and Rage, 1972 [3]

*Bransateryx* sp.

Figure 10

**Fig. 10:**
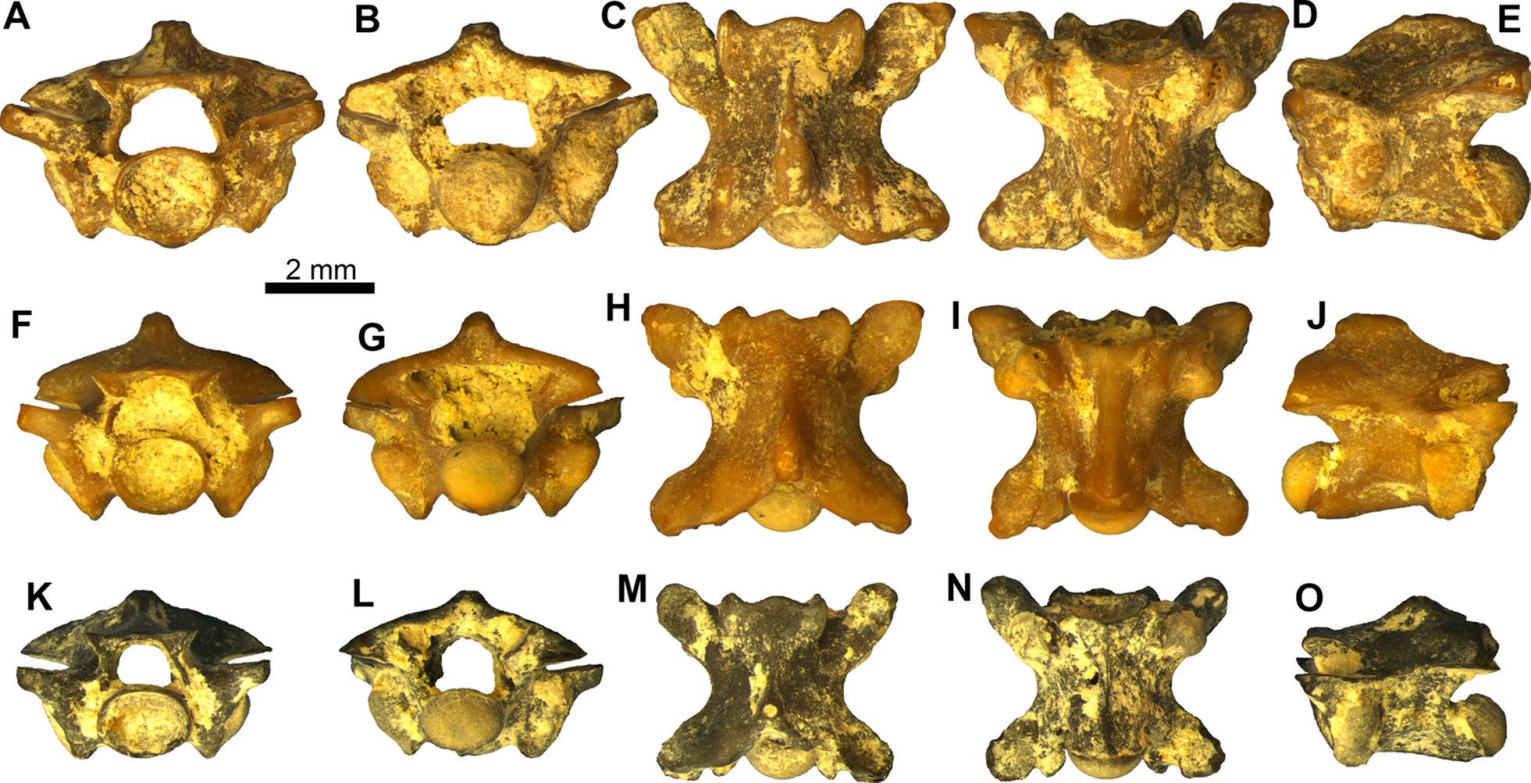
*Bransateryx* sp. trunk vertebrae: **A–E** trunk vertebra PIMUZ A/III 4653 in **A** anterior, **B** posterior, **C** dorsal, **D** ventral, and **E** left lateral views; **F–J** trunk vertebra PIMUZ A/III 4681 in **F** anterior, **G** posterior, **H** dorsal, **I** ventral, and **J** right lateral views; **K–O** trunk vertebra PIMUZ A/III 4654 in **K** anterior, **L** posterior, **M** dorsal, **N** ventral, and **O** left lateral views

**Material**: *Saint-Gérand-le-Puy*: two trunk vertebrae (PIMUZ A/III 4653 and PIMUZ A/III 4654); *Gannat*: a trunk vertebra (PIMUZ A/III 4681).

**Description**: All three vertebrae are rather small, with centrum lengths ranging between 3.0 and 3.8 mm (Fig. 10). In anterior view (Fig. 10A, F, K), the zygosphene is rather thin, with its dorsal level being almost straight, with the exception of PIMUZ A/III 4681, where this is slightly convex. The neural canal is large. The prezygapophyses are dorsally tilted. The cotyle is circular to slightly elliptical. In posterior view (Fig. 10B, G, L), the neural arch is rather depressed. The condyle is circular to slightly elliptical. In dorsal view (Fig. 10C, H, M), the neural spine is thicker towards the posterior portion of the neural arch. The zygosphene bears two prominent lateral lobes along with a less distinct medial one (which is more prominent in PIMUZ A/III 4681). The posterior median notch of the neural arch and the interzygapophyseal constriction are considerably deep. The prezygapophyses extend anterolaterally. The prezygapophyseal articular facets are large and oval. In ventral view (Fig. 10D, I, N), the haemal keel is crossing almost the whole mid-line length of the centrum, commencing anteriorly at the ventral level of the cotyle and terminating posteriorly prior to the condyle. The haemal keel is moderately thick in PIMUZ A/III 4653 and PIMUZ A/III 4681 but is considerably thinner in PIMUZ A/III 4654. The subcentral grooves are deep. The paradiapophyses are massive and are not clearly divided into diapophyseal and parapophyseal parts. The postzygapophyseal articular facets are large. In lateral view (Fig. 10E, J, O), the neural spine is rather short and its anteroposterior length is developed mostly towards the posterior half of the neural arch; it commences well below the level of the zygosphene and gradually increases in height. Lateral foramina are present below the level of the interzygapophyseal ridge. The subcentral ridges are convex. The haemal keel projects ventrally. All three vertebrae pertain to the posterior trunk portion of the column, judging from the width of the haemal keel (particularly in PIMUZ A/III 4653 and PIMUZ A/III 4681) and the depressed neural arch.

**Remarks**: As in all “erycines” (and unlike most other snakes), the main diagnostic vertebral features of *Bransateryx* lie in the caudal vertebrae [3, 116], elements which are absent from the Saint-Gérand-le-Puy collection. Nevertheless, the material described herein bears strong resemblance to trunk vertebrae previously referred to *Bransateryx*; these include trunk vertebrae of the only valid species, *Bransateryx vireti* Hoffstetter and Rage, 1972 [3], from its type locality (Coderet [MP 30], which is very near to Saint-Gérand-le-Puy), as well as a single trunk vertebra from the area of Saint-Gérand-le-Puy itself, described previously [3].

*Bransateryx* has been considered to pertain to erycine boids [3, 8, 59, 116]. However, it is now known, based on molecular data, that the traditional concept of erycines is paraphyletic, i.e., with the Old World erycines pertaining to a different group (Erycidae) than the New World ones (Charinainae). The latter group forms, together with Ungaliophiinae, the Charinaidae [115, 117, 118]. The most characteristic feature shared among Erycidae and Charinainae is the peculiar complex nature of the caudal vertebrae, which is nevertheless absent in the latter’s group closest relatives, i.e., the Ungaliophiinae [115, 118]. It is now known that charinaines were present in the European Eocene (i.e., *Rageryx* Smith and Scanferla, 2021 [118], from Messel) [115, 117, 118]; as such, it is currently not possible to assess the exact affinities of certain European Paleogene and early Neogene taxa that possess this kind of caudal vertebrae [115]. Accordingly, we follow the scheme of Smith and Georgalis [115], under which *Bransateryx* is placed into the informal group “erycines”.

## Discussion

### The diversity of squamates from the Saint-Gérand-le-Puy area

The different quarries of the Saint-Gérand-le-Puy complex have yielded a moderately diverse assemblage of earliest Miocene squamates. Taxa that had been documented with certainty from there include the gekkotans *Gerandogekko arambourgi* and *Euleptes gallica*, the lacertid *Lacerta poncenatensis*, indeterminate anguids, indeterminate lizards, the “erycine” *Bransateryx*, and indeterminate snakes [2, 3, 6, 23–27], while the slightly older, nearby site of Gannat yielded the lacertid *Pseudeumeces cadurcensis* [70]. To these, we now add the gallotiine lacertid *Janosikia*, at least one species of the anguine *Ophisaurus* (i.e., *Ophisaurus holeci*), plus more vertebral material of *Bransateryx*.

The identification of two different gekkotan species from the Saint-Gérand-le-Puy complex is of particular notice, especially when considering the rather poor Neogene fossil record of this group in Europe [119, 120]. In fact, the Saint-Gérand-le-Puy complex yielded the two out of five in total extinct Neogene gekkotan taxa known so far from Europe. *Gerandogekko arambourgi* is exclusively known from its type area in Saint-Gérand-le-Puy, while *Euleptes gallica* has been subsequently documented also from younger strata (Burdigalian) in Czech Republic [121]. It is further worth noting that both *Gerandogekko arambourgi* and *Euleptes gallica* are now considered as members of Sphaerodactylidae, a gekkotan group, which in Europe is currently represented only by a single species, *Euleptes europaea* (Gené, 1839) [116] (see [119, 122]).

Lacertids, the dominant and most speciose lizard group of extant European herpetofaunas, are also present in the Saint-Gérand-le-Puy fossil assemblage. *Lacerta poncenatensis* was established from its type locality, Poncenat in the Saint-Gérand-le-Puy complex [25]; this species has since been described also from the slightly coeval (MN 2a) locality of Amöneburg, Germany [11], while a similar form has been also described from the younger, Burdigalian (MN 4) locality of Oberdorf, Austria (*Lacerta* cf. *poncenatensis* of [123]). Augé [61] mentioned from Poncenat also another lacertid species, *Lacerta filholi*; however, without any published figure or description of that occurrence, it is impossible to assess this claim. Nevertheless, it has been recently suggested that *Lacerta poncenatensis* and *L. filholi* bear strong resemblance and they can be practically differentiated solely by their size and details in their tooth morphology at the posterior part of the dentary, features that are subject to strong intraspecific variability [123]. That being said, it could be probable that Augé’s [61] undescribed lacertid material could be conspecific with *L. poncenatensis*. Future research might even demonstrate that *L. poncenatensis* and *L. filholi* are synonyms, with the latter species then having nomenclatural priority. The large lacertid *Pseudeumeces cadurcensis* that has been described from Gannat [70], witnesses one of the youngest occurrences of *Pseudeumeces* Hoffstetter, 1944 [51], a genus that has been abundant and diverse during the Oligocene in Western and Central Europe [43, 70, 108]. We further documented here the presence of another lacertid genus in the Saint-Gérand-le-Puy complex, i.e., *Janosikia*. This new record provides a considerable geographic expansion for *Janosikia*, which was otherwise solely known from the type locality of its type species, *J. ulmensis*, in the early Miocene (MN 2a) of Ulm, Germany [12, 15], plus few younger indeterminate remains from the late early (MN 4) and middle Miocene (MN 5) of southern Germany [124] and the middle Miocene (MN 5) of Switzerland [125]. Recent phylogenetic analyses have placed *Janosikia* within the gallotiines, and precisely as the sister group of the extant insular genus *Gallotia* Boulenger, 1916 [126], from the Canary Islands [15, 127, 128], further demonstrating that gallotiines had already achieved a large size already by the early Miocene in continental Europe [15].

Two vertebrae from the Saint-Gérand-le-Puy complex that were described (but not figured) by Lydekker [24] who tentatively referred them to as *Placosaurus margariticeps*, were so far the only hint for the presence of anguids in the area. The new well preserved parietals described herein afford a more precise documentation of anguids in the locality, allowing the identification of *Ophisaurus holeci*, a species which was otherwise only known from the early Miocene (MN 3) of its type locality in Czech Republic, and few early (MN 2a and MN 3) and middle Miocene (MN 5 and MN 7/8) localities in southern Germany [14, 104, 106, 111]. This new record of *Ophisaurus holeci*, the first occurrence of the species from France, is therefore concordant with its large geographic and stratigraphic distribution. As discussed above, notably, *O. holeci* has been recently demonstrated by [14] to bear closest affinities with the Eocene genus *Ophisauriscus* rather than with the rest of *Ophisaurus* spp. Nevertheless, these authors chose not to recombine *O. holeci* into *Ophisauriscus* but selected to still treat the species within *Ophisaurus* [14]. If congeneric affinities of *O. holeci* with *Ophisauriscus* are indeed the case, that would mean a significant range extension of that genus from the Eocene to the Miocene. Accordingly, a more comprehensive revision and redescription of the type and only valid species of the former genus, *Ophisauriscus quadrupes* Kuhn, 1940 [13] from Geiseltal and Messel, is highly anticipated. The new vertebral material of *Ophisaurus* from Saint-Gérand-le-Puy does not allow a species level determination, as the genus was rather diverse during the early Miocene of Western and Central Europe [104, 106]*.*

The “erycine” snake *Bransateryx* was first documented from the Saint-Gérand-le-Puy complex by [3], with cranial material subsequently referred to it by [26]. The genus is abundant in the Oligocene of France and Germany [3, 5, 22, 26, 129]. It is probable that this earliest Miocene material from Saint-Gérand-le-Puy pertains to the sole valid species of the genus, *B. vireti*, which was typified from the nearby latest Oligocene locality of Coderet. Unfortunately, there are no caudal vertebrae in the Saint-Gérand-le-Puy complex that could confirm this with certainty.

The presence of the “anilioid” snake *Eoanilius* Rage, 1974 [64], in Montaigu-le-Blin has been mentioned [8], however, this was not described or figured. Indeed, Szyndlar and Rage ([8]:99) reported that its presence in that locality was “unquestionable”. The eventual description of that material is highly anticipated. In any case, such presence of *Eoanilius* in the Saint-Gérand-le-Puy complex would not be unexpected, as the genus is abundant across different early Miocene localities in Europe and Anatolia [5, 18, 130, 131].

Caenophidians are so far known in Saint-Gérand-le-Puy exclusively by the viperid described by [27]. The Saint-Gérand-le-Puy viperid has been considered to be of utmost importance, being treated as one of the oldest known viperids, and in particular the oldest known member of Viperinae [27, 68]. The only other viperine that could be potentially coeval or older is material from Weisenau (MN 1 or MN 2), Germany that has been referred to *Vipera* cf. *antiqua* [5]. *Vipera antiqua* Szyndlar, 1987 is originally typified from younger sediments, i.e., the Burdigalian (MN 4) of Dolnice, Czech Republic [116], and represents the sole extinct named species of the *Vipera aspis* complex [27, 65]. Indeterminate viperid material, that cannot be securely assigned to viperines or crotalines, has also been described from the Aquitanian of Germany [1, 9, 27].

Being one among a limited number of Aquitanian squamate bearing localities in Europe, Saint-Gérand-le-Puy has the potential to decipher important aspects in the evolution of lizard and snake assemblages in the earliest Miocene of the continent. We anticipate that the large collections from that area that have been accumulated across different European institutions, will shed more light on its, ever growing, taxonomic diversity.

### Virtual microanatomy and histology

For the dentary of *Janosikia*, seven growth marks could be counted, indicating an age range similar to extant *Gallotia* [132], its closest relative according to recent phylogenetic analysis [15]. Apart from a network of small tubes linking the alveolar canal with the pulp cavities and the external bone surface through small foramina, housing the mandibular vessels and the inferior alveolar nerve in the lower jaw (e.g., [133]), other histological features of the bone matrix are not visible in the scan.

Unfortunately, no growth marks were visible in the *Ophisaurus* parietals, but the scans revealed an extensive interconnected vascular network within the bones as well. The ornamentation pattern of anguine parietal bones has been shown to change throughout ontogeny, with vascular channels (or imprints on the dorsal bone surface) becoming successively overgrown by the ornamental bone tissue [33]. The ornamentation of anguine parietal bones, also due to their taxonomic value, has received quite some attention (e.g., [106]), including the use of CT scanning of bones (e.g., [14, 111]; see also recent study on ornamentation in gecko skulls [134]). On the other hand, the subsurface distribution and 3D connectivity of vascular channels, up to our knowledge, was not reported before in detail, although thin-sections of parietals of *Pseudopus apodus* were already processed and studied over 100 years ago [135]. At that time, Schmidt [135] interpreted the parietal as an inseparable composite of a dorsal, ornamented, and well-vascularized osteoderm, which sits on the smooth skull bone (the parietal). We reject this view for the *Ophisaurus holeci* specimens herein, because we interpret the successive increase of ornamentation as a direct continuation of the dorsal periosteal bone in anguine parietals, an interpretation that is backed up by the ontogenetic changes documented by [33].

We found that the parietals of *Ophisaurus holeci* are quite robust bones, with thick bone cortices ventrally and laterally, thus confirming the higher compactness values previously reported for some anguines [40]. We also show, however, that the more dorsally situated fine meshwork of channels of the *Ophisaurus holeci* parietal table connect with larger antero-posteriorly extending channels, and them opening up into larger cavities or sinuses underneath the smooth area posterior to the parietal table, giving the bone a more porous appearance. This particular vascular arrangement could indicate some thermoregulatory function (see [136] for potential functions of heavily vascularized ornamented bones in other tetrapods), where the ornamented parietal table could serve as a heat sink/collector and the larger sinuses could serve a storage or rapid drainage function of heated or cooled blood. However, comparative physiological studies using extant anguines such as *Ophisaurus* spp., *Pseudopus apodus*, and the fossils are needed to shed light on whether or what function the strong parietal vascularization has in these lizards.

## Conclusions

We conducted a detailed taxonomical documentation of a small collection of lizards and snakes remains from the earliest Miocene of Saint-Gérand-le-Puy, France. We documented novel occurrences for certain taxa. In addition, with the aid of micro-CT scanning, we attempted to decipher certain microanatomical and histological features, providing implications about their palaeophysiology. Detailed anatomical studies coupled with novel microanatomical and virtual histology data, could serve as invaluable resources for the study of squamates from the earliest Miocene, a time interval that was quintessential for the evolution of lizards and snakes of Europe and the onset of modern herpetofaunas.

## Supplementary Information

**Additional file 1:** Video of the 3D model of left dentary PIMUZA/III 4656 of *Janosikia* sp.

**Additional file 2:** Video of the 3D model of the left dentary PIMUZA/III 4656 of *Janosikia* sp. showing internal vascularization.

**Additional file 3:** Video of the 3D model of the parietal PIMUZA/III 4626 of *Ophisaurus holeci.*

**Additional file 4:** Video of the 3D model of the parietal PIMUZA/III 4626 of *Ophisaurus holeci *showing internal vascularization.

**Additional file 5:** Video of the 3D model of the parietal PIMUZA/III 4627 of *Ophisaurus holeci.*

**Additional file 6:** Video of the 3D model of the parietal PIMUZA/III 4627 of *Ophisaurus holeci *showing internal vascularization.

## Data Availability

All specimens described and figured herein are permanently curated at the collections of PIMUZ. The CT scans and 3D models of the three specimens which were micro-CT scanned are deposited at the Morphosource repository (https://www.morphosource.org/): CT image series of the dentary PIMUZ A/III 4656 of *Janosikia* sp. (https://doi.org/10.17602/M2/M365352); 3D model of the dentary PIMUZ A/III 4656 of *Janosikia* sp. (https://doi.org/10.17602/M2/M368405); CT image series of the parietal PIMUZ A/III 4626 of *Ophisaurus holeci* (https://doi.org/10.17602/M2/M365341); 3D model of the parietal PIMUZ A/III 4626 of *Ophisaurus holeci* (https://doi.org/10.17602/M2/M368585); CT image series of the parietal PIMUZ A/III 4627 of *Ophisaurus holeci* (https://doi.org/10.17602/M2/M365346); 3D model of the parietal PIMUZ A/III 4627 of *Ophisaurus holeci* (https://doi.org/10.17602/M2/M368581).

## Abbreviation

PIMUZ: Palaeontological Institute and Museum of the University of Zurich, Zurich, Switzerland
